# Supplementary material for: Nucleoporin Elys attaches peripheral chromatin to the nuclear pores in interphase nuclei
Source: Commun Biol. 2024 Jun 29;7:783. doi: 10.1038/s42003-024-06495-w (PMC11217421; doi:10.1038/s42003-024-06495-w)
Supplement: Supplementary file 1 — Supplementary Information [file 42003_2024_6495_MOESM1_ESM.pdf]

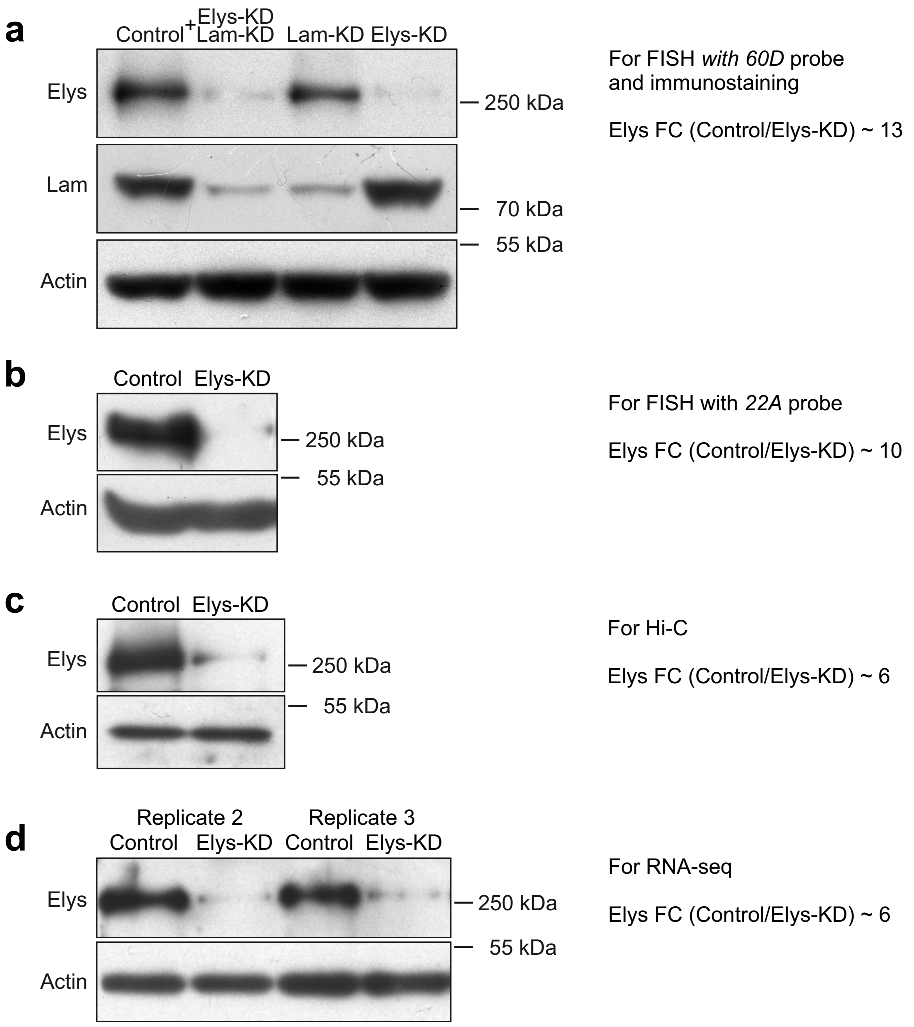

Supplementary Figure 1 An efficiency of Elys-KD. a–d Western-blot analysis of protein extracts from control, Elys-KD, LamKD or Elys-KD + Lam-KD S2 cells probed by anti-Elys, anti-Lam or anti-Actin (loading control) antibodies. Quantification of depletion efficiency was performed in *ImageJ*. In (**a**–**c**) protein extracts from two replicates were combined before loading.

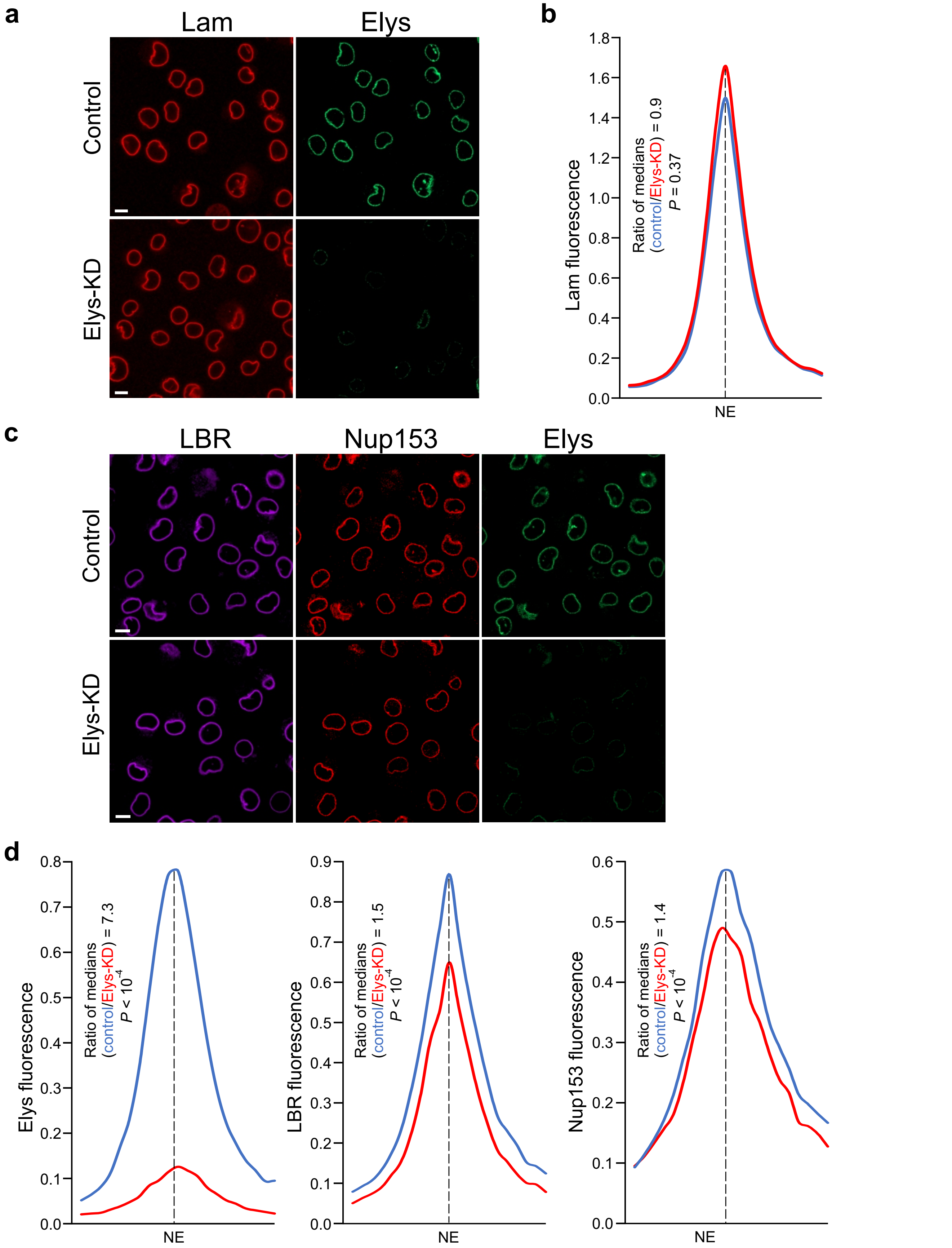

Supplementary Figure 2 Elys-KD in S2 cells does not notably impair Lam, LBR or Nup153 localization at the NE. **a,c** Confocal images of control or Elys-KD S2 cells after immunostaining with anti-Lam (red) and anti-Elys (green) antibodies (**a**), or with anti-LBR (violet), anti-Nup153 (red) and anti-Elys (green) antibodies (**c**). Scale bars 5 µm. **b,d** *ImageJ* quantification of average fluorescence intensity of Lam (**b**), or Elys, LBR and Nup153 (**d**) (normalized on average Dapi fluorescence) across the NE in Elys-KD S2 cells (two replicates, n = 75 (**b**) or n =40 (**d**) each) and control (two replicates, n = 80 (**b**) or n = 40 (**d**) each). Ratio of median values is indicated. *P* values were estimated in a M-W *U*-test.

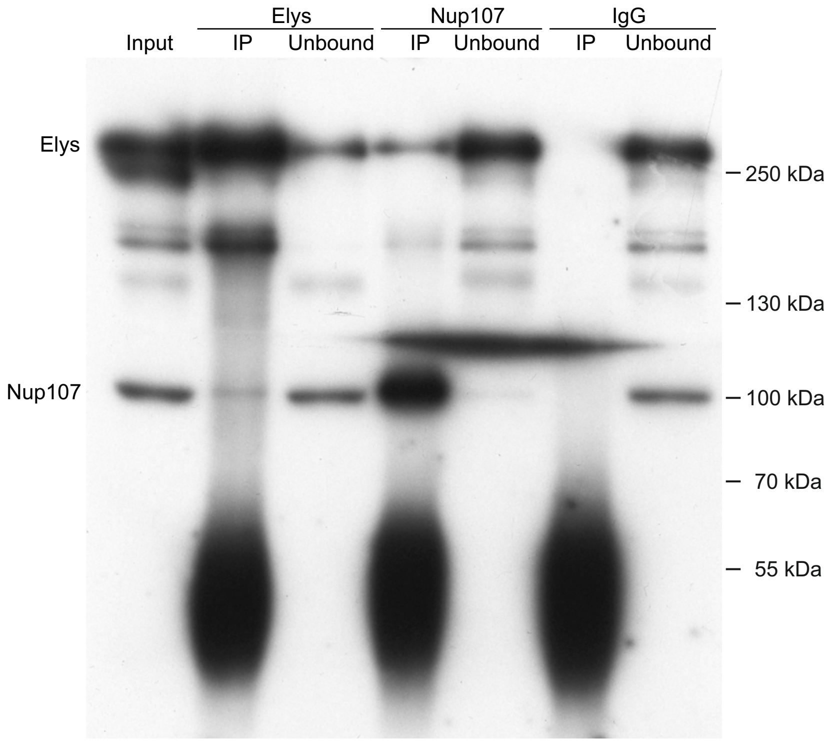

Supplementary Figure 3 (related to Figure 1e) Western-blot showing co-immunoprecipitation results. Western-blot analysis of protein extracts from S2 cells after co-immunoprecipitation with anti-Nup107 or anti-Elys antibodies. The blot was first probed with anti-Nup107 antibodies and then by anti-Elys antibodies. Strong bands at the bottom of the gel represent immunoglobulin heavy chains. Some non-specific bands revealed by anti-Elys antibodies are also present.

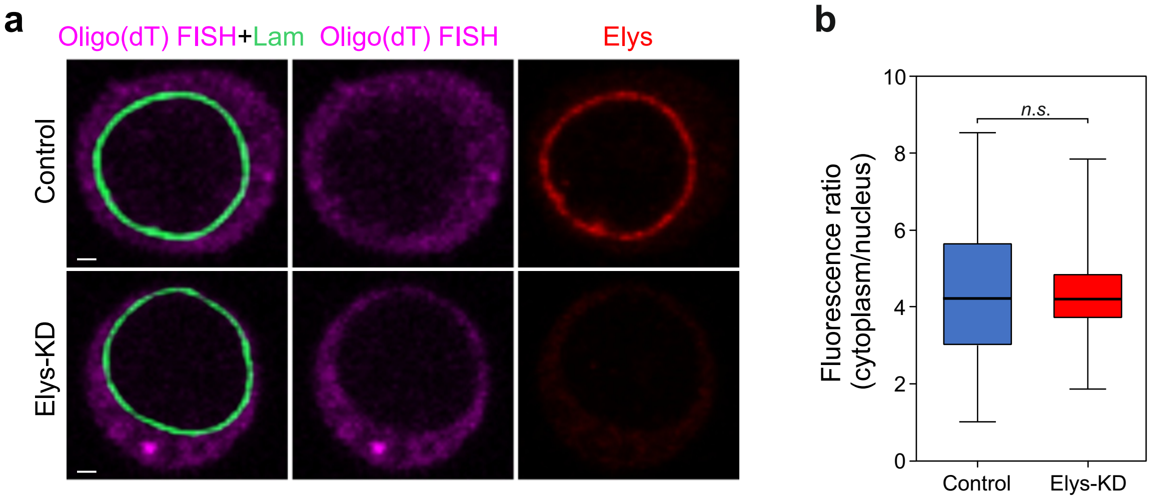

Supplementary Figure 4 Upon Elys-KD, mRNA export is not impaired. a Confocal images of control or Elys-KD S2 cells after oligo(dT) FISH (violet) and immunostaining with anti-Lam (green) and anti-Elys (red) antibodies. Scale bar 1 μm. **b** Box-plots showing ratios of fluorescence intensities (in cytoplasm to nucleus) for oligo(dT) FISH. Fluorescence in the 40 control and Elys-KD cells (from two replicates) was quantified using *ImageJ*. *P* value was estimated in a M-W *U*-test. n.s. - non-significant ($P > 0.05$). See Fig. 6c legend for description of box-plot elements.

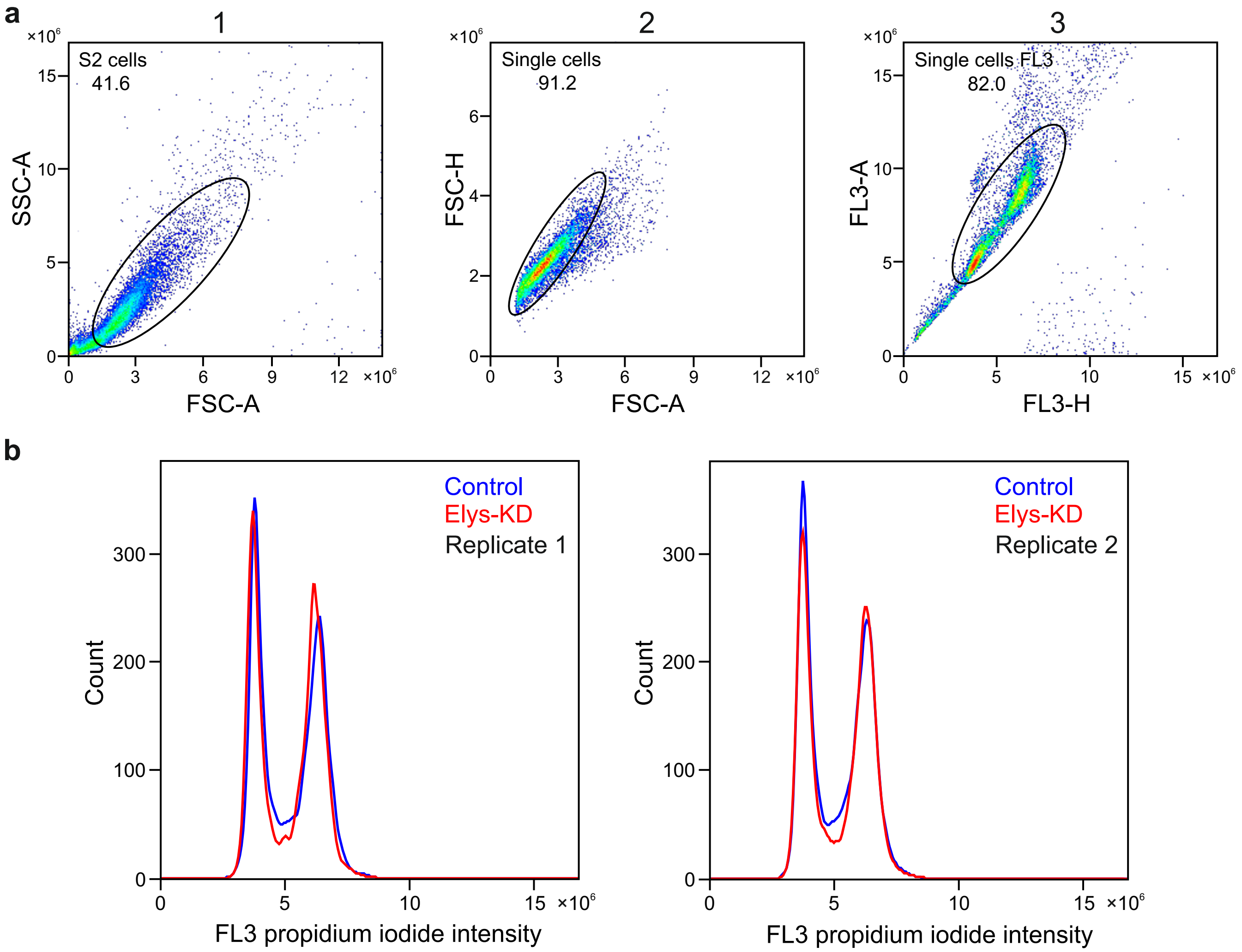

Supplementary Figure 5 Upon Elys-KD, cell cycle progression of S2 cells is only barely affected. (**a**) Gating strategy for flow cytometry. 1. FSC-area/SSC-area gate was used to remove debris and dead cells. 2. FSC-area/FSC-height gate was used to identify single cells. 3. FL3-height/FL3-area gate was used to determine single propidium iodide-stained cells. (**b**) Flow cytometry analysis of control (blue curve) and Elys-KD (red curve) S2 cells after DNA staining with propidium iodide. Two replicates are indicated.

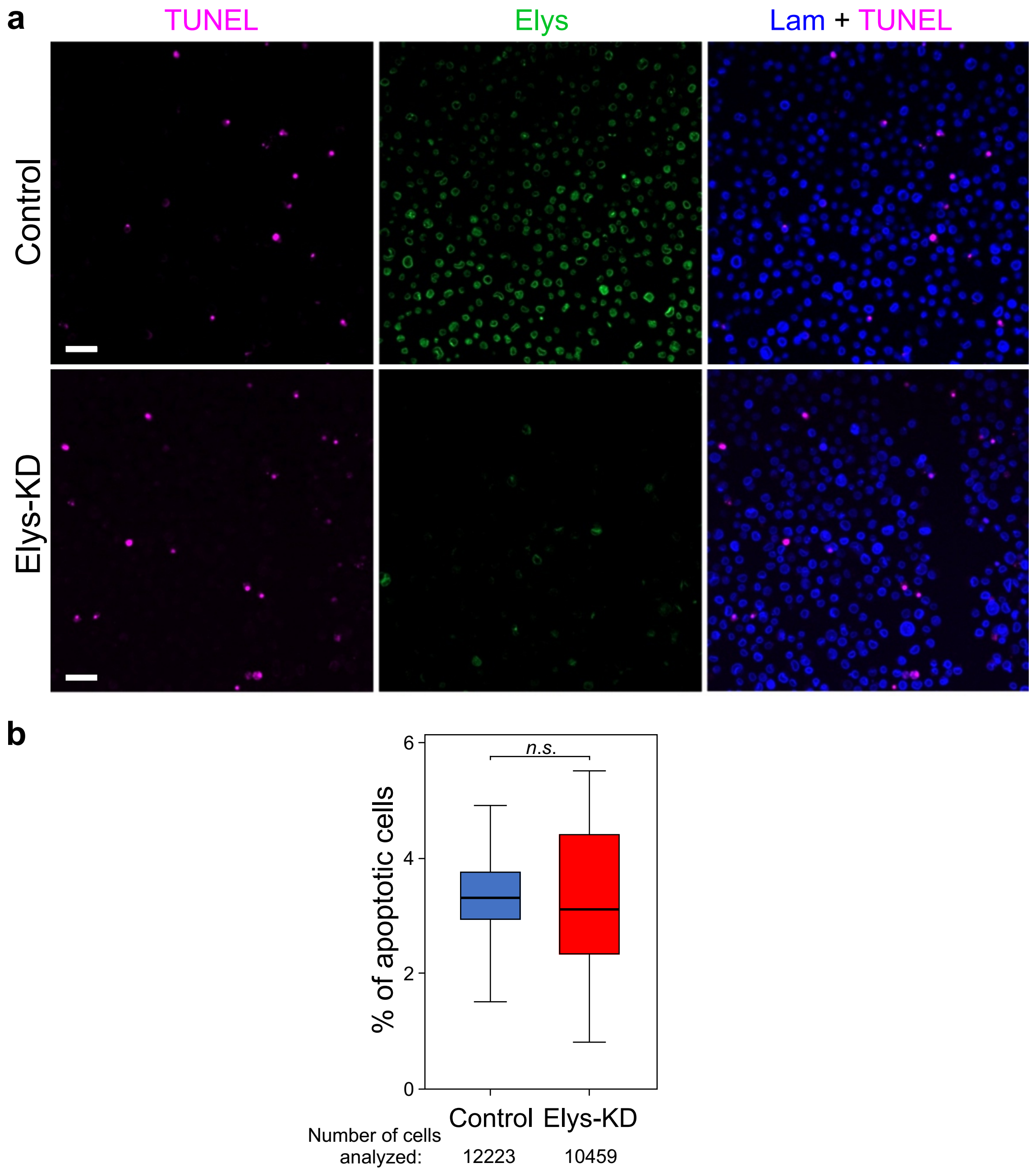

Supplementary Figure 6 TUNEL assay did not detect an increase in the proportion of apoptotic cells upon Elys-KD. a Confocal image of control or Elys-KD S2 cells stained with TUNEL assay (violet), as well as with anti-Elys (green) and anti-Lam (blue) antibodies. Scale bars 30 μm. **b** Box-plots showing percentage of apoptotic cells in control or Elys-KD S2 cells. *P* value was estimated in a M-W *U*-test. n.s. - non-significant ($P > 0.05$). See Fig. 6c legend for description of box-plot elements.

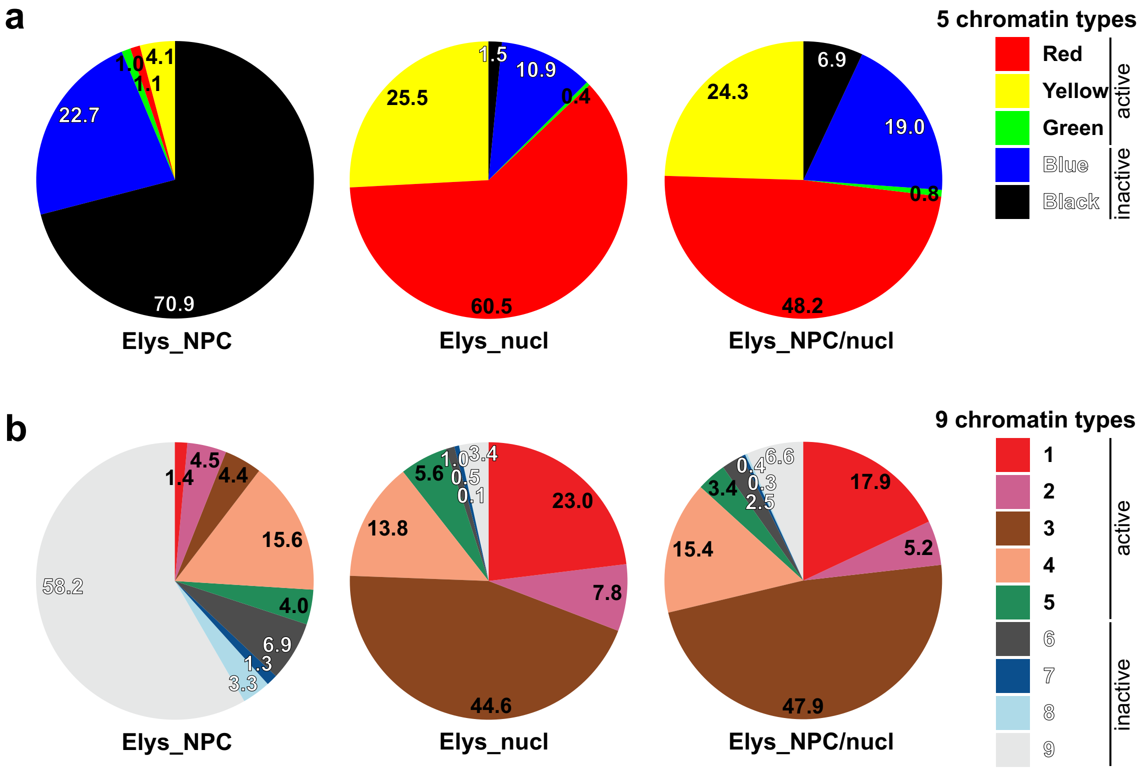

Supplementary Figure 7 Elys_NPC and Elys_nucl sites mostly overlap with inactive and active chromatin types, respectively. a,**b** Pie charts showing percentage of overlap between Elys_NPC, Elys_nucl or Elys_NPC/nucl sites with chromatin domains identified according to 5-state chromatin model in Kc167 cells (**a**), or according to 9-state chromatin model in S2 cells (**b**).

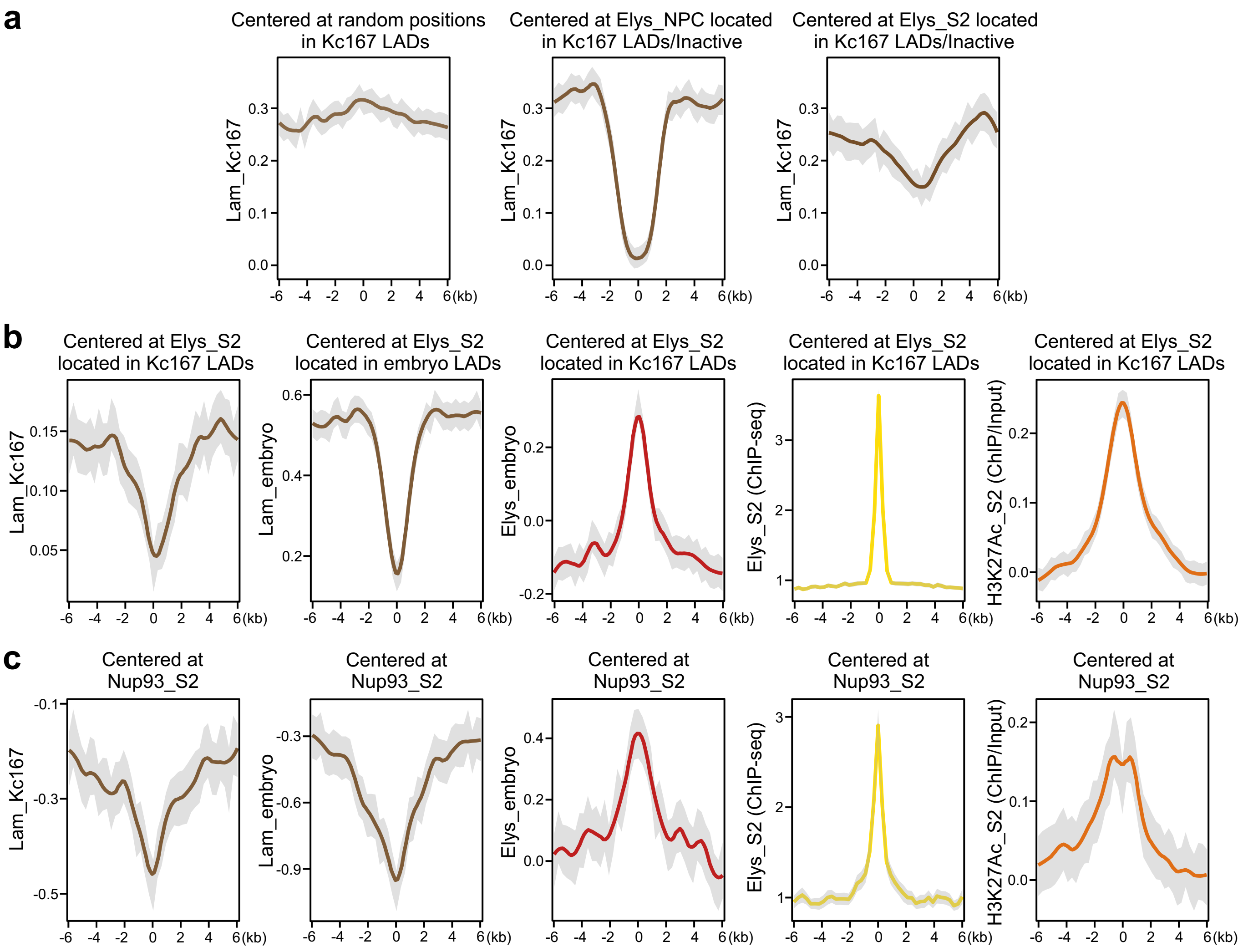

Supplementary Figure 8 Weak dips in Lam profile at positions of Elys ChIP-seq peaks from S2 cells. a–c Averaged Lam_Kc167, Lam_embryo, Elys_embryo, Elys_S2 and H3K27Ac profiles centered at randomly chosen positioned within Kc167 LADs, or centered at Elys_NPC sites located within Kc167 LADs overlapped with inactive chromatin (states 6-9), or centered at Elys_S2 ChIP-seq sites located within Kc167 LADs overlapped with inactive chromatin (states 6-9) (**a**), or centered at Elys_S2 ChIP-seq sites located within Kc167 or embryo LADs (±2 kb from LAD boundaries) (**b**), or centered at Nup93_S2 ChIP-seq sites (**c**).

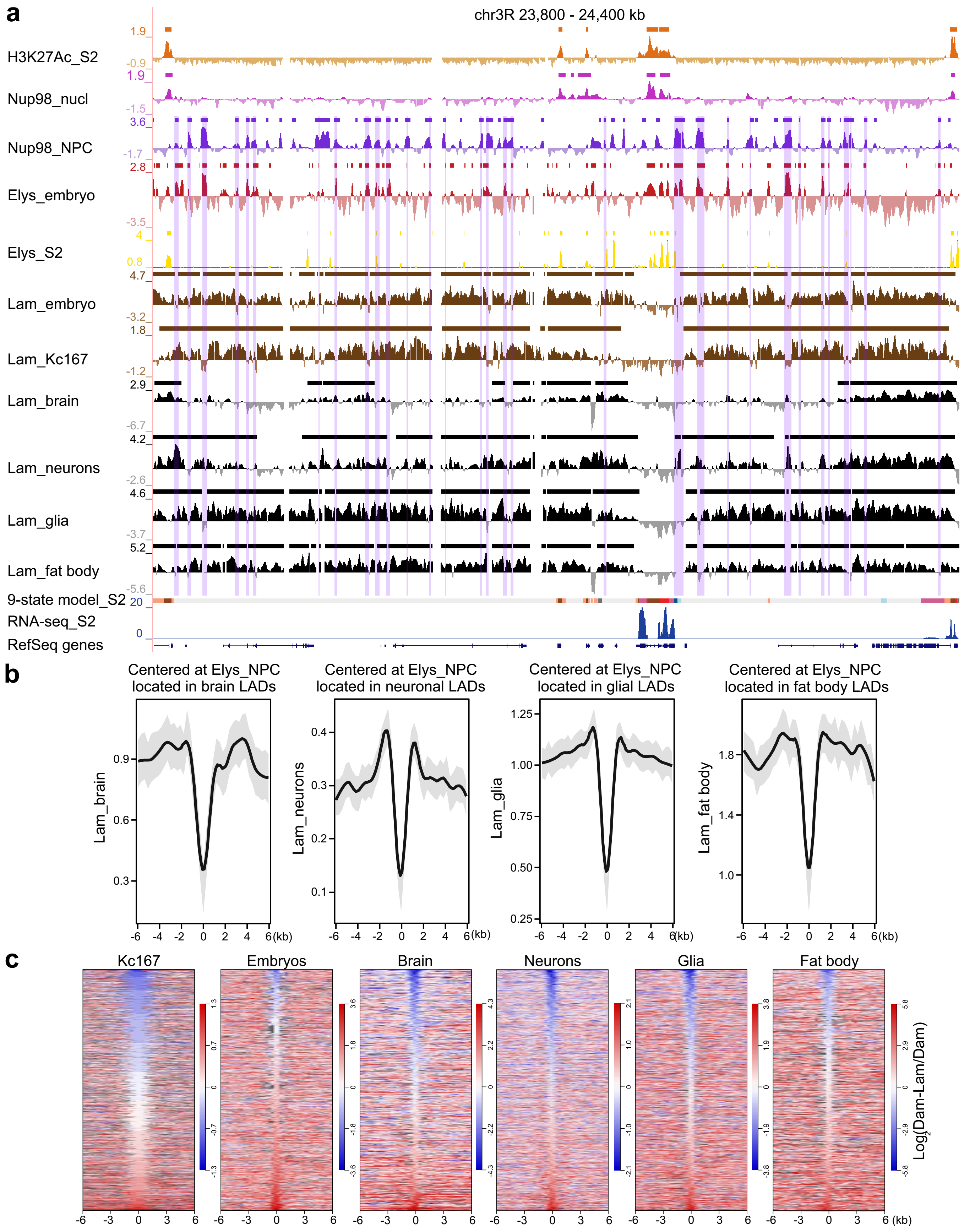

Supplementary Figure 9 The dips in Lam profile are revealed at positions of Elys_NPC sites during cell differentiation. a Screenshot from UCSC genome browser showing H3K27Ac (orange), Nup98_nucl (pink), Nup98_NPC (violet), Elys_embryo (red), Elys_S2 (yellow), Lam_embryo and Lam_Kc167 (brown) profiles, as well as Lam_brain, Lam_neurons, Lam_glia and Lam_fat body (black) profiles for the representative region of chromosome 3R. The corresponding domains are provided as the rectangles over profiles. 9-state chromatin model, RNA-seq in control S2 cells and RefSeq genes are indicated below. The overlapped regions between Elys_embryo and Nup98_NPC domains containing Elys_NPC peaks are outlined by translucent rectangles (they mostly coincide with the dips in Lam profiles during cell differentiation). **b** Averaged Lam_brain, Lam_neurons, Lam_glia and Lam_fat body profiles centered at Elys_NPC sites, located within LADs, from the corresponding cell type. **c** Heatmaps of Lam profiles centered at Elys_NPC sites, located within LADs, from the corresponding cell type sorted from minimal (top) to maximal (bottom) values at the central bins. Rows where central bins had zero value either in Dam-Lam or in Dam profiles were removed. Bins with zero value of either Dam-Lam or Dam in other locations are marked by grey color.

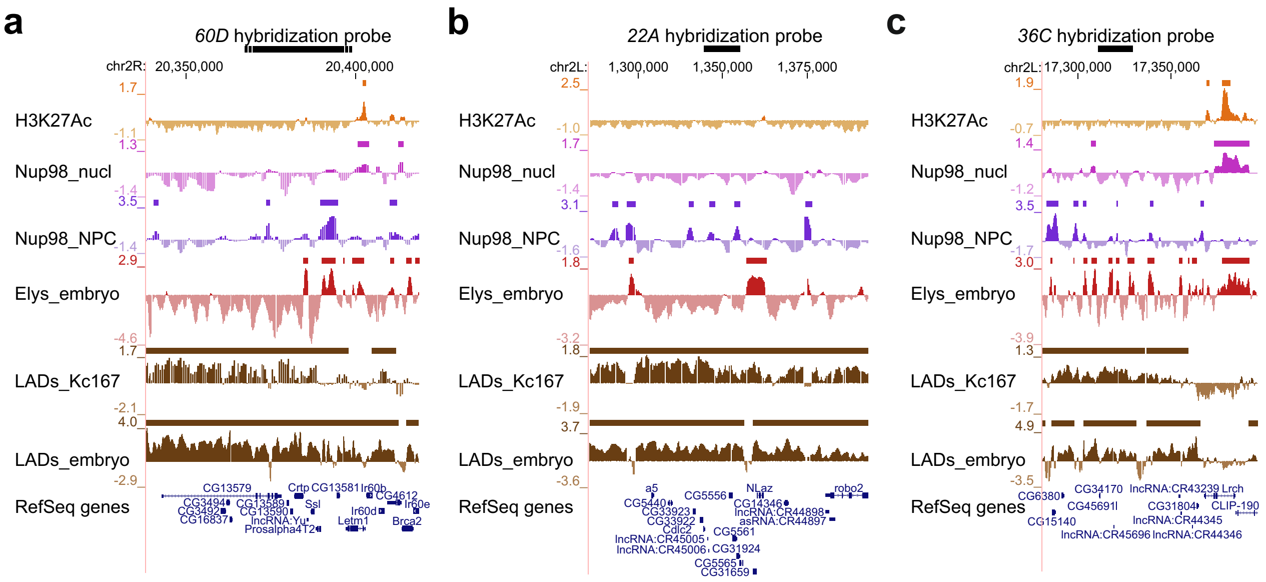

Supplementary Figure 10 ***60D*****,** ***22A*** **and** ***36C*** **hybridization probes and/or their nearby regions contain Elys_NPC sites. a**–**c** Screenshots from UCSC genome browser showing H3K27Ac (orange), Nup98_nucl (pink), Nup98_NPC (violet), Elys_embryo (red), Lam_Kc167 and Lam_embryo (brown) profiles, as well as the corresponding domains (rectangles over profiles) for the *60D* (**a**), *22A* (**b**), and *36C* (**c**) regions. RefSeq genes are indicated below. Hybridization probes are indicated by black rectangles on the top of the panels. Exact boundaries of the *60D* probe are unknown.

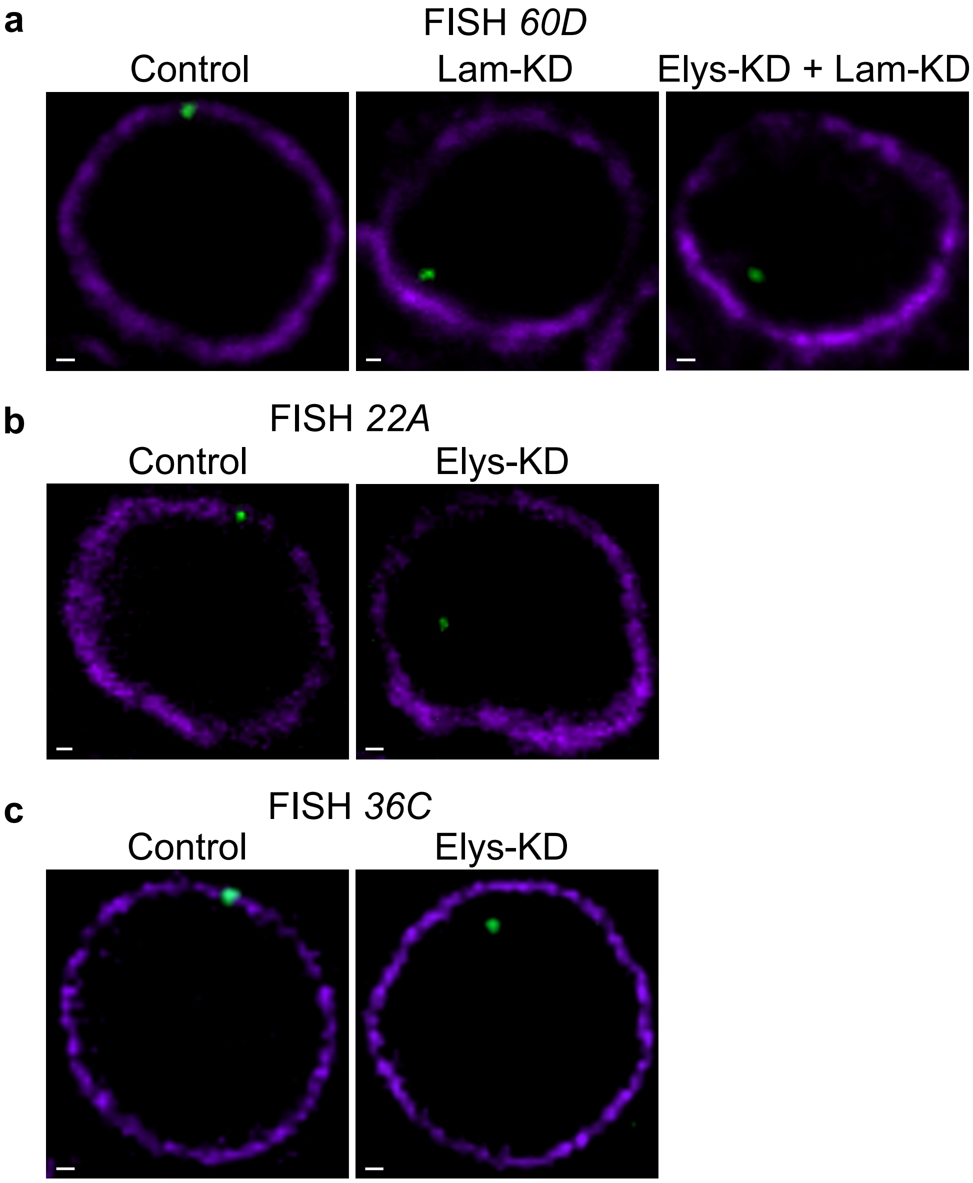

Supplementary Figure 11 Representative confocal images of FISH signals. a–**c** Confocal images of FISH signals (green) detected by the probe for *60D* region (**a**), *22A* region (**b**), or *36C* region (**c**) in nuclei stained with anti-LBR (violet) (**a**,**b**) or anti-Lam (violet) (**c**) antibodies in Elys-KD, Lam-KD, Elys-KD plus Lam-KD, or control S2 cells. Scale bars 0.5 μm.

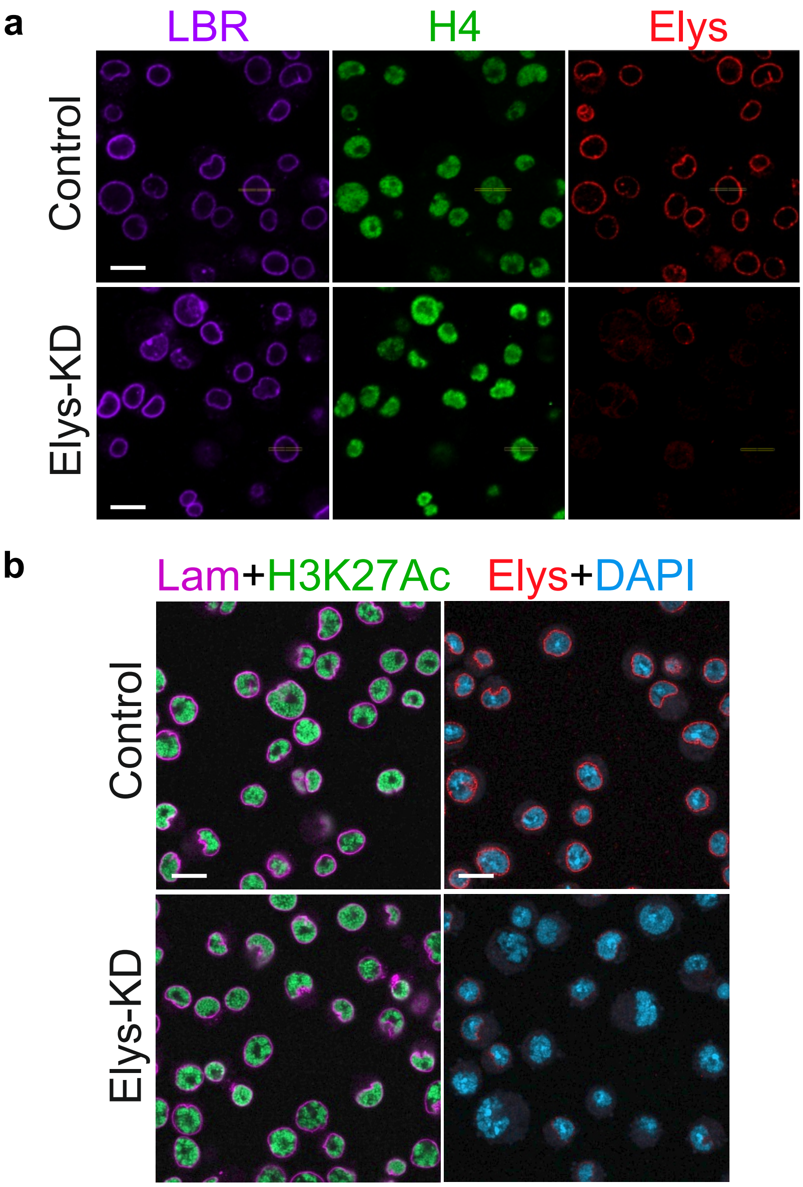

Supplementary Figure 12 Analysis of chromatin distribution in nuclei upon Elys-KD. a Staining of control and Elys-KD cells with anti-LBR (violet), anti-histone H4 (green) and anti-Elys (red) antibodies. **b** Staining of control and Elys-KD cells with anti-Lam (pink), anti-H3K27Ac (green) and anti-Elys (red) antibodies. Scale bars 10 µm.

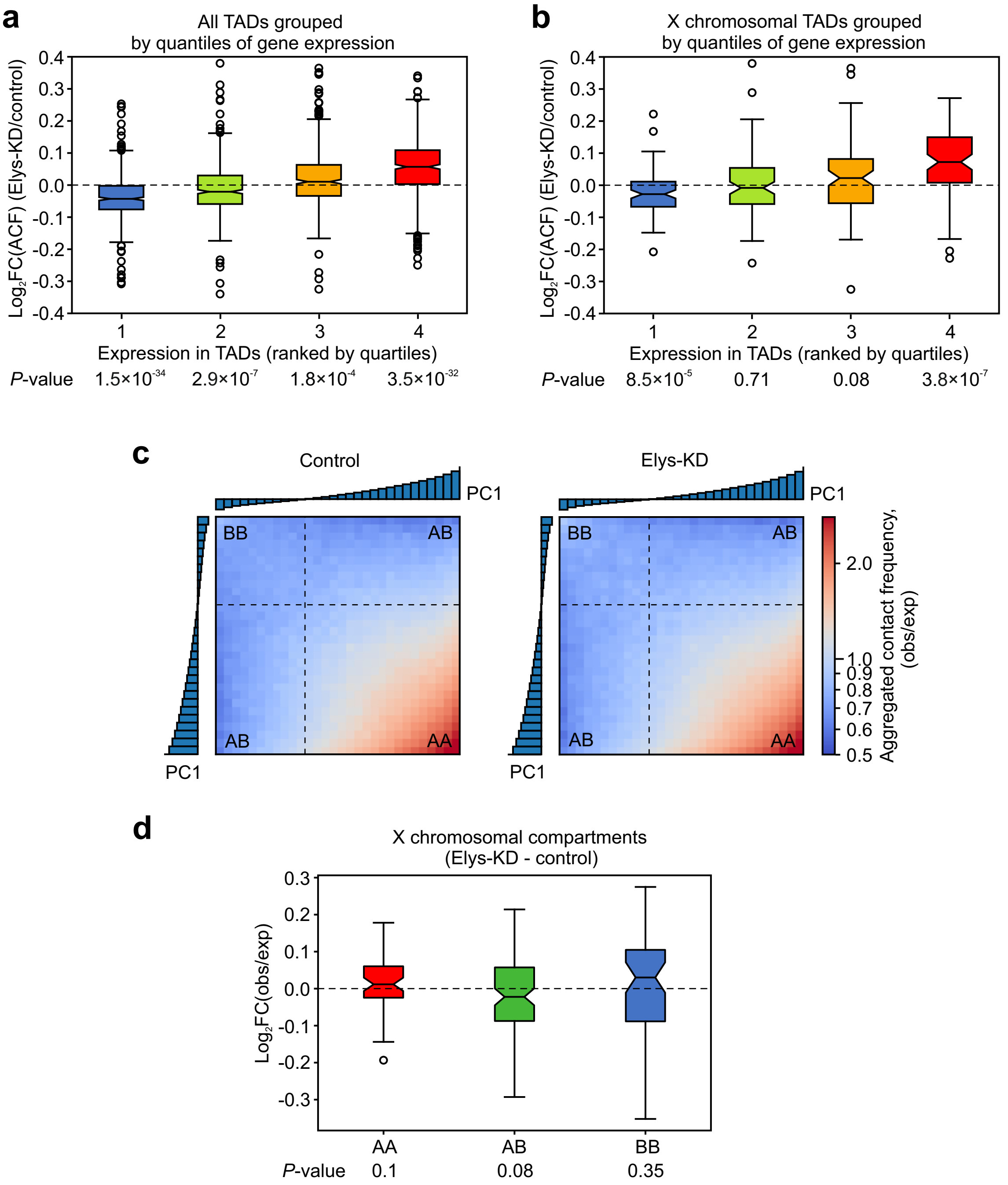

Supplementary Figure 13 Active chromatin became more compact, whereas inactive - less compact upon loss of Elys in S2 cells. a,**b** Log_2 fold-change (FC) of ACF (Elys-KD/control) in all TADs (**a**) or in the X chromosomal TADs (**b**) ranked by quartiles of total gene expression within them (according to RNA-seq data (in RPM) in control S2 cells, where 1st quartile corresponds to the lowest, and 4th quartile - to the highest gene expression). *P*-values were estimated in a Wilcoxon signed-rank test. **c** Saddle plots showing values of aggregated contact frequency for the intra-chromosomal contacts in autosomes in control (left panel) and Elys-KD (right panel) cells ranked by PC1 values. **d** Box-plots showing subtraction of aggregated contact frequency (Elys-KD minus control) log_2FC(observed/expected) for only the X chromosome within active (AA), inactive (BB), and between active and inactive (AB) chromatin compartments. *P*-values were estimated in a Wilcoxon signed-rank test. See Fig. 6c legend for description of box-plot elements.

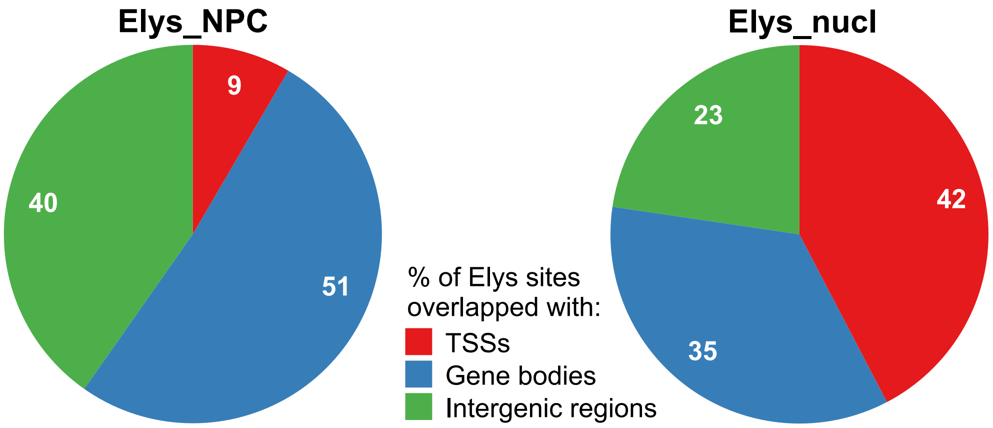

Supplementary Figure 14 Pie chart showing percentage of Elys binding sites overlapped with genes or intergenic regions.

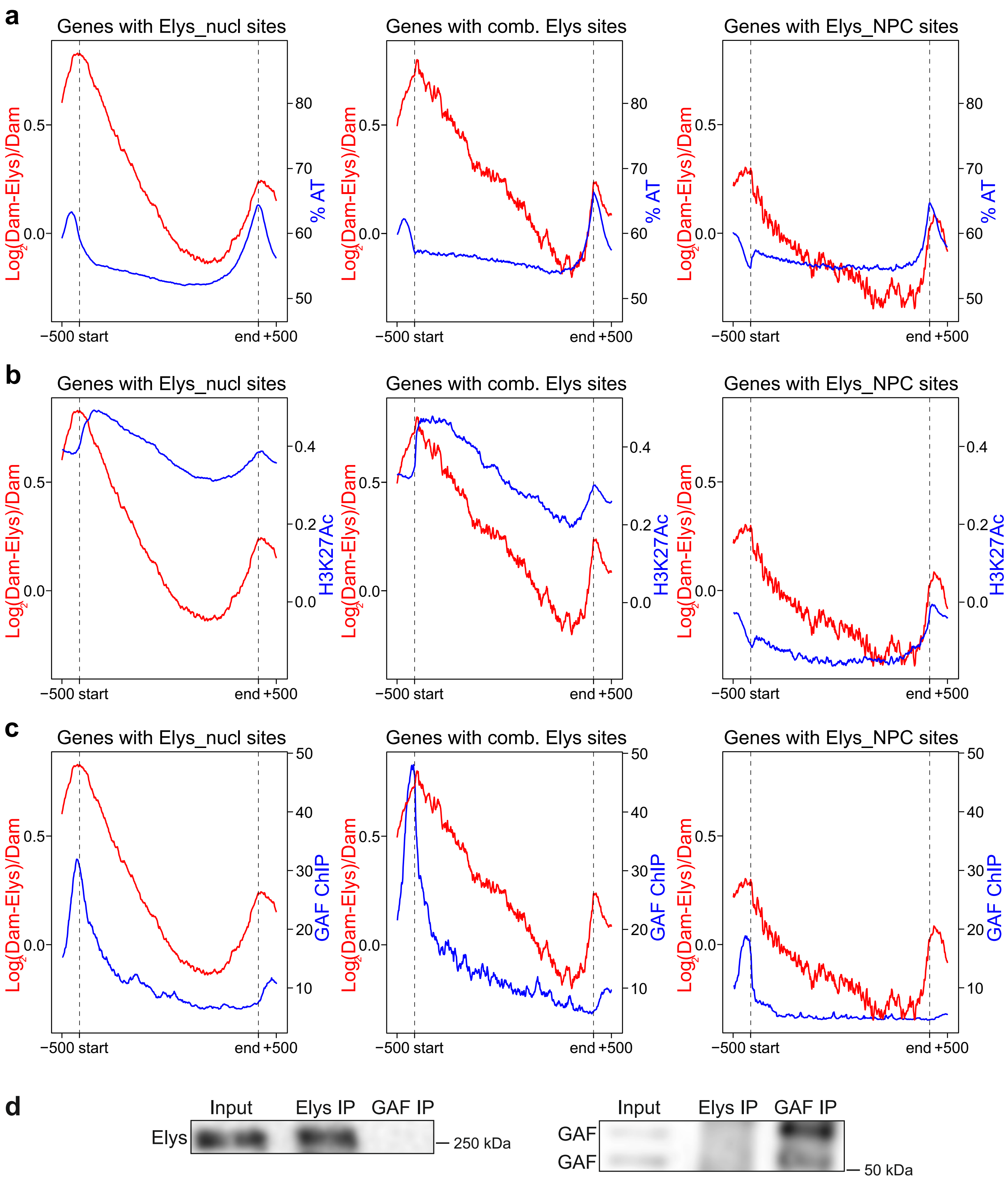

Supplementary Figure 15 Elys is enriched at the 5'- and 3'-ends of genes. a–**c** Metagene profiles for Elys (red) and A/T content (blue) (**a**), Elys (red) and H3K27Ac (blue) (**b**), Elys (red) and GAF (blue) (**c**) over genes containing Elys_nucl, Elys_NPC sites or combinations of these sites. **d** Western-blot analysis does not reveal co-immunoprecipitation of Elys and GAF. Westerns are stained with anti-Elys (left panel) or anti-GAF (right panel) antibodies. Anti-GAF antibodies detect two GAF isoforms. IP/input ratio 1:4.5.

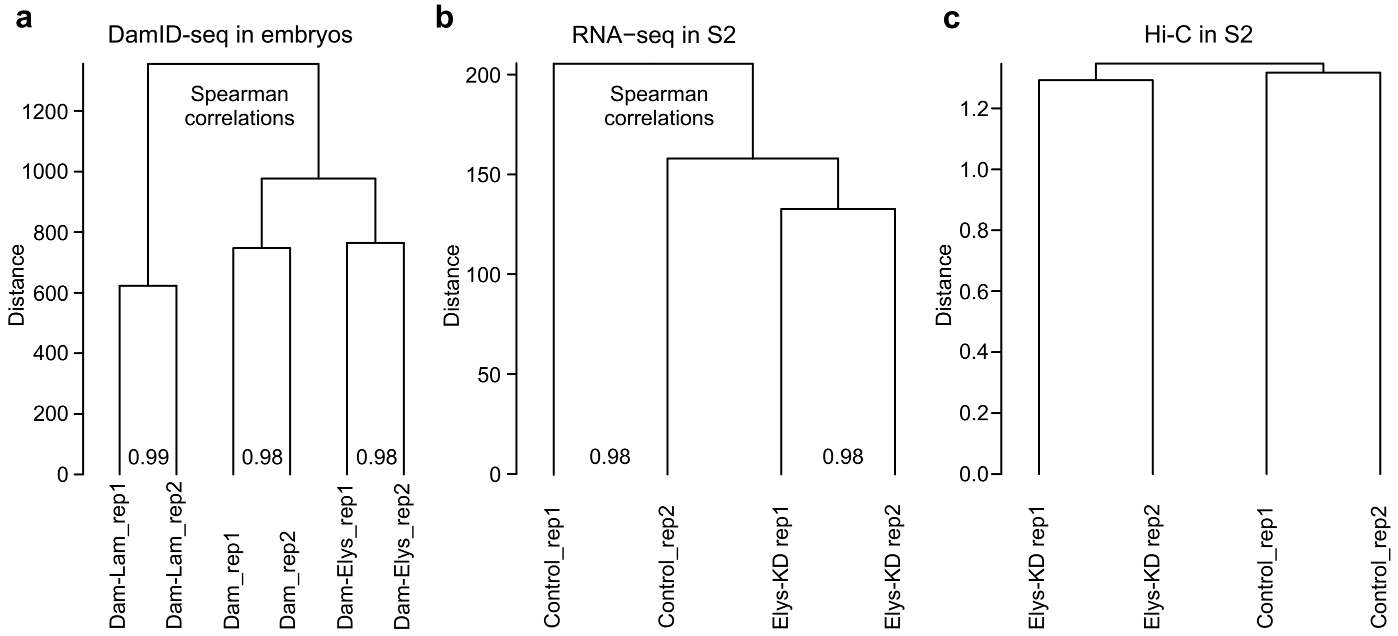

Supplementary Figure 16 Replicates clustering. a–**c** Replicates for DamID-seq (**a**), RNA-seq (**b**) and Hi-C (**c**) are highly correlated according to the Spearman correlation coefficient (**a**,**b**), or to the stratum-adjusted correlation coefficient (SCC) (**c**).
