# Supplementary material for: Nucleoporin Elys attaches peripheral chromatin to the nuclear pores in interphase nuclei
Source: Commun Biol. 2024 Jun 29;7:783. doi: 10.1038/s42003-024-06495-w (PMC11217421; doi:10.1038/s42003-024-06495-w)
Supplement: Supplementary file 2 — Description of Additional Supplementary Files [file 42003_2024_6495_MOESM2_ESM.pdf]

Description of Additional Supplementary Files

File name: Supplementary Data 1

Description: Quantification of fluorescence intensity of Lam, Mab414, Elys, LBR and Nup153 at the nuclear envelope in control and Elys-KD S2 cells (related to Fig. 1 and Supplementary Fig. 2)

File name: Supplementary Data 2

Description: TUNEL assay analysis in control and Elys-KD S2 cells (related to Supplementary Fig. 6)

File name: Supplementary Data 3

Description: Elys_embryo, Elys_NPC, Elys_nucl and Elys_NPC/nucl sites in late embryos (related to Fig. 3 and Supplementary Fig. 8)

File name: Supplementary Data 4

Description: LADs in late embryos (related to Fig. 3)

File name: Supplementary Data 5

Description: FISH analysis in control and ElysKD S2 cells (related to Fig. 5)

File name: Supplementary Data 6

Description: Quantification of histone H4 and H3K27Ac fluorescence intensity across a nucleus in control and Elys-KD S2 cells (related to Fig. 5)

File name: Supplementary Data 7

Description: Volume of nuclei in control and Elys-KD S2 cells analyzed in IMARIS (related to Fig. 5)

File name: Supplementary Data 8

Description: TADs in control and Elys-KD S2 cells (related to Fig. 6 and Supplementary Fig. 13)

File name: Supplementary Data 9

Description: Expresion in control and Elys-KD S2 cells by RNA-seq (related to Fig. 7)

File name: Supplementary Data 10

Description: Primers for PCR amplification

File name: Supplementary Data 11

Description: NGS statistics
