# Supplementary material for: Nucleoporin Elys attaches peripheral chromatin to the nuclear pores in interphase nuclei
Source: Commun Biol. 2024 Jun 29;7:783. doi: 10.1038/s42003-024-06495-w (PMC11217421; doi:10.1038/s42003-024-06495-w)
Supplement: Supplementary file 14 — Reporting summary [file 42003_2024_6495_MOESM14_ESM.pdf]

nature portfolio

Corresponding author(s): Yuri Shevelyov
Last updated by author(s): Jun 6, 2024

Reporting Summary

Nature Portfolio wishes to improve the reproducibility of the work that we publish. This form provides structure for consistency and transparency in reporting. For further information on Nature Portfolio policies, see our Editorial Policies and the Editorial Policy Checklist.

Statistics

For all statistical analyses, confirm that the following items are present in the figure legend, table legend, main text, or Methods section.

n/a	Confirmed	
☐	☒	The exact sample size (n) for each experimental group/condition, given as a discrete number and unit of measurement
☐	☒	A statement on whether measurements were taken from distinct samples or whether the same sample was measured repeatedly
☐	☒	The statistical test(s) used AND whether they are one- or two-sided<br>*Only common tests should be described solely by name; describe more complex techniques in the Methods section.*
☒	☐	A description of all covariates tested
☒	☐	A description of any assumptions or corrections, such as tests of normality and adjustment for multiple comparisons
☒	☐	A full description of the statistical parameters including central tendency (e.g. means) or other basic estimates (e.g. regression coefficient) AND variation (e.g. standard deviation) or associated estimates of uncertainty (e.g. confidence intervals)
☒	☐	For null hypothesis testing, the test statistic (e.g. F, t, r) with confidence intervals, effect sizes, degrees of freedom and P value noted<br>*Give P values as exact values whenever suitable.*
☒	☐	For Bayesian analysis, information on the choice of priors and Markov chain Monte Carlo settings
☒	☐	For hierarchical and complex designs, identification of the appropriate level for tests and full reporting of outcomes
☒	☐	Estimates of effect sizes (e.g. Cohen's d, Pearson's r), indicating how they were calculated

Our web collection on statistics for biologists contains articles on many of the points above.

Software and code

Policy information about availability of computer code

Data collection	In-house scripts are available at Zenodo: https://zenodo.org/record/7808900#.ZDBCLvZBxPY.
Data analysis	In-house scripts are available at Zenodo: https://zenodo.org/record/7808900#.ZDBCLvZBxPY.

For manuscripts utilizing custom algorithms or software that are central to the research but not yet described in published literature, software must be made available to editors and reviewers. We strongly encourage code deposition in a community repository (e.g. GitHub). See the Nature Portfolio guidelines for submitting code & software for further information.

Data

Policy information about availability of data

All manuscripts must include a data availability statement. This statement should provide the following information, where applicable:

- Accession codes, unique identifiers, or web links for publicly available datasets
- A description of any restrictions on data availability
- For clinical datasets or third party data, please ensure that the statement adheres to our policy

Raw and processed DamID-seq, RNA-seq, and Hi-C data were deposited in the NCBI Gene Expression Omnibus (GEO) under the accession numbers GSE219152 (for DamID-seq, RNA-seq) and GSE218886 (for Hi-C). The numerical source data behind the graphs in the paper can be found in Supplementary Data. All other data and materials, including plasmid DNA, that support the findings of this study are available from the corresponding authors upon reasonable request. Uncropped/unedited blot image related to Fig. 1e is presented in Supplementary Fig. 3. The link for UCSC Genome Browser showing Elys-DamID profile https://

genome.ucsc.edu/cgi-bin/hgTracks?db=dm3&lastVirtModeType=default&lastVirtModeExtraState=&virtModeType=default&virtMode=0&nonVirtPosition=&position=chr2R%3A16166667-16833333&hgsid=2292689452_aXCaEPy5C7QaMaomtYGbtb1oGayN.

Human research participants

Policy information about studies involving human research participants and Sex and Gender in Research.

Reporting on sex and gender	Not applicable.
Population characteristics	Not applicable.
Recruitment	Not applicable.
Ethics oversight	Not applicable.

Note that full information on the approval of the study protocol must also be provided in the manuscript.

Field-specific reporting

Please select the one below that is the best fit for your research. If you are not sure, read the appropriate sections before making your selection.

☒ Life sciences ☐ Behavioural & social sciences ☐ Ecological, evolutionary & environmental sciences

For a reference copy of the document with all sections, see nature.com/documents/nr-reporting-summary-flat.pdf

Life sciences study design

All studies must disclose on these points even when the disclosure is negative.

Sample size	No sample-size calculation was performed.
Data exclusions	No data were excluded from the analysis.
Replication	All experiments were performed at least in two biological replicates.
Randomization	The permutation test with random reshuffling of genomic sites and genes within Drosophila genome was applied to estimate P-values.
Blinding	Analysis of FISH and immunostaining data was done with the blind experimental setup.

Reporting for specific materials, systems and methods

We require information from authors about some types of materials, experimental systems and methods used in many studies. Here, indicate whether each material, system or method listed is relevant to your study. If you are not sure if a list item applies to your research, read the appropriate section before selecting a response.

Materials & experimental systems

n/a	Involved in the study	
☐	☒	Antibodies
☐	☒	Eukaryotic cell lines
☒	☐	Palaeontology and archaeology
☒	☐	Animals and other organisms
☒	☐	Clinical data
☒	☐	Dual use research of concern

Methods

n/a	Involved in the study	
☒	☐	ChIP-seq
☐	☒	Flow cytometry
☒	☐	MRI-based neuroimaging

Antibodies

Antibodies used	Rabbit polyclonal anti-H3K27Ac antibodies, Abcam, ab4729;<br>Murine monoclonal anti-histone H4 antibodies, Abcam, ab31830;<br>Murine monoclonal anti-lamin Dm0 antibodies ADL84 were provided by Paul Fisher (Stony Brook University School of Medicine);<br>Murine monoclonal anti-lamin Dm0 antibodies ADL67 were provided by Paul Fisher (Stony Brook University School of Medicine);<br>Rabbit polyclonal anti-Elys antibodies were generated in our laboratory;

	Guinea-pig polyclonal anti-LBR antibodies were provided by Georg Krohne (Julius-Maximilians-Universität Würzburg);<br>Murine monoclonal anti-beta Actin antibodies, Abcam, ab8224;<br>Rabbit polyclonal anti-Nup107 antibodies were provided by Valerie Doye (Institut Jacques Monod);<br>Mouse monoclonal anti-Nup153 antibodies were provided by Daria Kopytova (Institute of Gene Biology, RAS);<br>Rabbit polyclonal anti-GAF antibodies were provided by Maksim Erokhin (Institute of Gene Biology, RAS);<br>Chicken anti-CenpA antibodies were provided by Gary Karpen (University of California, Berkeley);<br>Murine monoclonal Mab414 antibodies, Abcam ab24609;<br>Rabbit polyclonal alpha-Tubulin, Abcam ab18251.
Validation	Broad reactivity of anti-H3K27Ac or anti-histone H4 antibodies is confirmed by the manufacturer:<br>https://www.abcam.com/products/primary-antibodies/histone-h3-acetyl-k27-antibody-chip-grade-ab4729.html<br>https://www.abcam.com/products/primary-antibodies/histone-h4-antibody-mabcam-31830-chip-grade-ab31830.html;<br>anti-Elys antibodies were tested in our previous study (Ilyin et al. 2017, doi: 10.1093/nar/gkx355);<br>all other antibodies were tested in the corresponding studies (references are provided in the text of the manuscript).

Eukaryotic cell lines

Policy information about cell lines and Sex and Gender in Research

Cell line source(s)	S2 cell line was obtained from the Institute of Molecular Genetics collection.
Authentication	None of the cell lines used were authenticated.
Mycoplasma contamination	S2 cell line is negative for mycoplasma contamination.
Commonly misidentified lines<br>(See ICLAC register)	Commonly misidentified cell lines were not used in this study.

Flow Cytometry

Plots

Confirm that:

☒ The axis labels state the marker and fluorochrome used (e.g. CD4-FITC).

☒ The axis scales are clearly visible. Include numbers along axes only for bottom left plot of group (a 'group' is an analysis of identical markers).

☒ All plots are contour plots with outliers or pseudocolor plots.

☒ A numerical value for number of cells or percentage (with statistics) is provided.

Methodology

Sample preparation	S2 cells were fixed with 70% cold ethanol, incubated with RNase A and PI and then analyzed by flow cytometry.
Instrument	BD Accuri™ C6 Plus Flow Cytometer
Software	FlowJo
Cell population abundance	~10000 single cell events
Gating strategy	FCS-Area/SSC-Area gate was used to remove debris and dead cells. FSC-Area/FSC-Height gate was used to identify single events. FL3-Height/FL3-Area gate was used to determine single propidium iodide-stained events.

☒ Tick this box to confirm that a figure exemplifying the gating strategy is provided in the Supplementary Information.
